# Neurological Disorders in Primary Sjögren's Syndrome

**DOI:** 10.1155/2012/645967

**Published:** 2012-03-05

**Authors:** Gabriel J. Tobón, Jacques-Olivier Pers, Valérie Devauchelle-Pensec, Pierre Youinou

**Affiliations:** ^1^EA Immunologie et Pathologie, Université de Bretagne Occidentale et Centre Hospitalier Universitaire de Brest, BP 824, F2969 Brest, France; ^2^Laboratory of Immunology, Brest University Medical School, BP 824, 2969 Brest, France

## Abstract

Sjögren's syndrome is an autoimmune disease characterized by an autoimmune exocrinopathy involving mainly salivary and lacrimal glands. The histopathological hallmark is periductal lymphocytic infiltration of the exocrine glands, resulting in loss of their secretory function. Several systemic manifestations may be found in patients with Sjögren's syndrome including neurological disorders. Neurological involvement ranges from 0 to 70% among various series and may present with central nervous system and/or peripheral nervous system involvement. This paper endeavors to review the main clinical neurological manifestations in Sjögren syndrome, the physiopathology, and their therapeutic response.

## 1. Epidemiology of Neurological Involvement in Sjögren's Syndrome

Sjögren's syndrome (SS) is a common autoimmune disease (AID) characterized by an autoimmune exocrinopathy [1] involving mainly salivary and lacrimal glands. The histopathological hallmark is periductal lymphocityc infiltration of the exocrine glands, resulting in loss of their secretory function. This disease occurs alone as primary SS (pSS), or in a background of connective tissue diseases as secondary SS (sSS). Even though keratoconjunctivitis sicca (resulting from the involvement of lacrimal glands) and xerostomia (resulting from that of salivary glands) are usually prominent, SS presents as a multifaceted condition with a broad variety of clinical manifestations (i.e., fatigue, arthralgias, Raynaud's phenomenon, interstitial pneumonias, lymphadenopathy, vasculitic urticaria, purpura, renal tubular acidosis, and neurological involvement) and biological abnormalities of B lymphocytes manifests as hypergammaglobulinemia; production of anti-SSA and anti-SSB autoantibodies and of rheumatoid factor; and an increased risk of non-Hodgkin's B-cell lymphoma (NHL) [2, 3]. 

This polymorphism accounts for the delay in the diagnosis. As a consequence, there is very likelihood that the prevalence of the disease is far higher than previously estimated [4]. European Community Study Group on diagnostic criteria for SS (2002) is used to classify patients with the disease [5].

Neurological involvement in SS may be manifested in the central nervous system (CNS) and/or peripheral nervous system (PNS). The prevalence of neurological manifestations ranges between 0 and 70% according to the investigators and depending on the recruitments of their clinics, but in general, such complications occur in about 20% of patients [6–12]. This impressive heterogeneity may be explained by the medical department where patients are recruited (i.e., internal medicine versus neurology) [8], the diagnosis criteria for pSS used (before 2002), or the definition of specific neuropathies and the diagnostic test performed to classify the neurological involvement (mainly in asymptomatic patients). Notably, series published before year 2002 included some patients as considered as suffering from pSS without histology and/or antibody evidence. Comparison between these series is impeded by the heterogeneity in the diagnostic criteria.

To illustrate this concern, in a series by Lafitte et al. [8], neurological manifestations in pSS were analyzed in two cohorts from two medical departments (25 patients from internal medicine and 11 patients from neurology department). Neurological involvement was found in 40% of patients from the internal medicine department. PNS involvement was present in 4 of 25 patients from the internal medicine group, whereas, in the neurology department, there were 10 of 11 patients (mainly axonal sensorimotor/sensory polyneuropathy). CNS involvement occurred in 7/25 patients from the internal medicine department and 4/11 from neurology. Cognitive dysfunction was the most frequent CNS finding. Thus, these results confirmed that neurological involvement in SS varies according to medical department where patients are evaluated.

Selection of patients in the different series is other matter of concern. Most of these series have been constructed retrospectively. For example, Mori et al. [11] reported 92 patients evaluated by neurological symptoms, but the majority of patients (93%) were diagnosed with pSS after neuropathy. Patients were evaluated between 1985 and 2004. Thus, part of patients was diagnosed with the criteria proposed by the Diagnostic Committee of Health and Welfare of Japan (1999) [13]. On the other hand, Gøransson et al. [12] in a cross-sectional study evaluated PNS in 62 pSS patients applying the American-European classification criteria. In this series, 27% of patients presented neuropathy after clinical examination, and 55% had abnormal conduction studies.

Neurological manifestations may precede the sicca symptoms in 40 to 93% of the cases [8, 14]. As described by Mori et al. [11], 93% of patients were diagnosed with pSS after neuropathy symptoms appeared. Patients with pSS and neurological involvement are older than patients without neurological implication [9, 10].

pSS-associated neurological main manifestations are listed in Table 1. PNS involvement in pSS is well characterized, manifested mainly as axonal polyneuropathies (sensory and sensorimotor), trigeminal neuropathy, and small-fiber neuropathy. Distal axonal sensory or sensorimotor polyneuropathy accounts for over 50% of cases of PNS involvement [6, 7, 15]. On the other hand, CNS manifestations are heterogeneous, manifested as focal or diffuse involvement. Most series reported that PNS involvement is more common than CNS disease. However, Delalande et al. reported the same frequency of central and peripheral nervous system involvements [15].

Other aspect to analyze is the severity of evaluated patients. Most of the previous studies have been conducted at reference centers, thus probably patients seen in these studies have a more severe disease. Lopate et al. [16] showed the prevalence of neuropathy in pSS in an outpatient setting. In the outpatient context, they evaluated 22 pSS patients and 10 controls for evidence of neuropathy. Isolated small-fiber neuropathy was found in 45% of cases and none of controls. Large-fiber dysfunction was similar between the two groups. This study highlights the importance of subclinical neuropathy present in many pSS patients that may lead to disability related to painful distal paresthesias and also the clinical differences according to the patient setting.

## 2. Pathophysiology

The pathogenic mechanisms responsible for most forms of neurological involvement in pSS are unknown. To explain this involvement, many hypothesis have been considered. Three pathogenic factors may explain the CNS disorders. The first hypothesis is the direct infiltration of the CNS by mononuclear cells [17]. Bakchin et al. [17] reported a patient with ataxia, oculomotor paralysis, seizures, and a large lymphocytic infiltrate at postmortem examination. The second hypothesis is the vascular involvement. The vascular injury may be related to the presence of antineuronal antibodies and anti-Ro antibodies [18]. Finally, Alexander [19–21] suggest that the underlying mechanism of CNS lesion in pSS is the ischemia secondary to small vessel vasculitis. 

**Figure 1:**
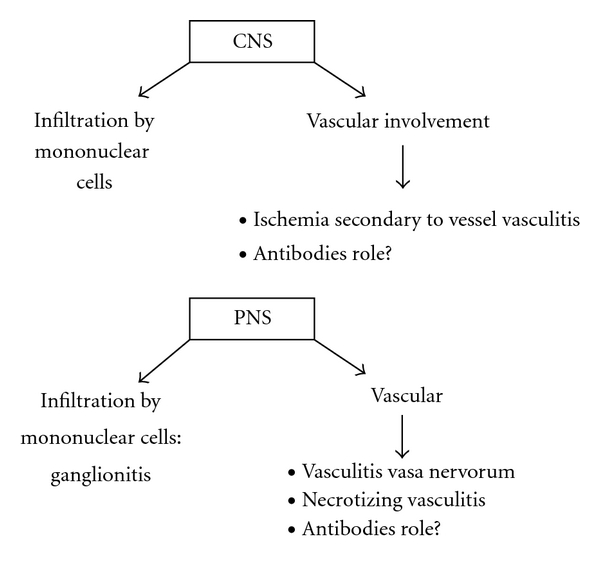
Pathophysiological mechanism implicated in the development of central and peripheral nervous system manifestations in primary Sjögren's syndrome.

Several mechanisms are suggested for the development of the involvement of PNS in pSS patients. Vascular or peripheral inflammatory infiltrates with or without necrosis may be found [14, 22]. Vasculitis of the vasa nervorum has also been proposed as pathogenic mechanism in PNS involvement [14]. However, others studies have not replicated these findings [15, 23]. In the case of motor neuropathy, necrotizing vasculitis may be found. Lymphocytic infiltration of the dorsal ganglia has been found in some cases of sensory neuronopathy [24]. Antineuronal antibodies have also been described in patients with PNS involvement [25], but the pathological role of these antibodies remains unknown. Antibodies against the type 3 muscarinic receptor have also been described in pSS. These antibodies have shown to be functional, and they are able to inhibit neuron-mediated contraction throughout the gastrointestinal tract. Thus, these antibodies may eventually explain part of the broader autonomic dysfunction found in pSS patients [26].  Figure 1 summarizes the main pathophysiological mechanisms. 

## 3. Central Nervous System Involvement

CNS involvement has not been as well defined as the PNS involvement. Thus, CNS involvement in pSS is controversial, and its prevalence ranges from 0% to 68% [15], according to different series [27–29]. García-Carrasco et al. reported only 1% of CNS involvement (4 patients in a cohort of 400 pSS patients) [29]. SNC involvement varies from diffuse compromise, manifested as cognitive deficits or meningoencephalitis, to focalized compromise, with spinal involvement or optic myelitis. The diagnostic is more difficult compared to PNS involvement, due to unspecific symptoms.

Alexander et al. [18] described CNS manifestations in 20% of pSS patients. The same group [6] showed that 63% of patients presenting CNS involvement had PNS manifestations. Escudero et al. [30] reported that headache is the main CNS complication in pSS. In addition, subclinical tissue injury may be determined by magnetic resonance imaging (MRI). This method also permits to determine the extension and severity of CNS involvement [27, 31].

Due to high variation in clinical symptoms and signs derived from CNS involvement, some authors propose that these manifestations in pSS are a fortuity association and the link between CNS manifestations and pSS is not well characterized.

### 3.1. Focal Involvement

Focal encephalic involvement is the main CNS manifestation in pSS [6, 15]. These focal disorders can include motor and sensory loss with hemiparesis, aphasia, dysarthria, seizures, movement disorders, and cerebellar syndrome. Their onset may be acute of insidious or even in a recurring pattern that resembles to multiple sclerosis. Some criteria such as older age, PNS or cranial nerve involvement, spinal cord MRI lesions spanning multiple segments, and cerebral MRI showed cortical brain lesions are characteristic of pSS involvement and rarely seen in multiple sclerosis [27, 28].

Spine cord disorders can include acute or chronic progressive myelopathies, lower motor neuron disease, or neurogenic bladder [15, 32, 33]. Spine complications may be associated with encephalic involvement. In the series by Lafitte et al. [8], myelopathies are reported in 3 of 11 patients with SNC involvement. The clinical picture is often characterized by transverse myelitis [32–34]. Although rare in pSS, acute and chronic myelopathies are frequently severe and life-threatening. These manifestations usually respond poorly to treatment with corticosteroids. Immunosuppressive treatment with cyclophosphamide and steroids has shown some efficacy in patients with progressive disease (see Section 7).

Subacute transverse myelitis with high signal on T2 weighted images and abnormal cerebrospinal fluid (CSF) study (increased protein level and cell count) is a rare but well-described complication in pSS patients [35, 36].

Optic neuropathies have been also described in pSS [37]. This manifestation can be asymptomatic. Alexander [38] reported seven cases of retrobulbar optic neuropathy in pSS patients. Four asymptomatic patients were diagnosed by visual evoked potentials. 

Sanahuja et al. [31] described a case of a pSS patient with a large tumefactive brain lesion, who responded well to oral corticosteroid treatment. This lesion, although rarely reported, has to be considered in pSS patients. Differential diagnosis includes lymphoma, glioma, abscesses, metastasis, progressive multifocal leukoencephalopathy, and disseminated encephalomyelitis.

### 3.2. Diffuse Involvement

CNS involvement can be diffuse, presenting encephalopathy, cognitive disfunction, dementia, psychiatric abnormalities, and aseptic meningoencephalitis [39–41]. This last complication is characterized by abnormal CSF, with lymphocytic cells and proteins.

Cognitive disturbances of variable severity have been described in pSS patients without mood disorders [8]. Lafitte el al. reported 8 from 36 pSS patients with cognitive dysfunction, characterized by frontal executive dysfunction, impairment in attention control, intellectual decline, and deterioration of instrumental abilities. Cognitive impartment is not correlated with CSF abnormalities or MRI findings [42, 43]. Malinow et al. [44] described 25 psychiatric abnormalities in 40 pSS patients. Of 16 patients undergoing cognitive function testing, 7 presented mild memory impairment with attention and concentration deficits. Belin et al. [45] evaluated 14 pSS patients with brain MRI, brain 99 m Tc-SPECT, and neuropsychological testing. In this series, all patients presented neuropsychological abnormalities, mostly frontal lobe syndrome and memory problems. The neurological involvement was associated with SPECT abnormalities, but not MRI imaging results. Ferreiro et al. reported a patient with diffuse angiographic changes, supporting that an ischemic mechanism caused by CNS vasculitis may be responsible for the clinical presentation in some patients [46].

In conclusion, these studies show the wide range of CNS manifestations that could be associated with pSS. Also, it is important to recognize cognitive problems, which are common in pSS, and cognitive evaluation is a sensible tool sensible to diagnose CNS compromise.

## 4. Peripheral Nervous System Involvement

As described in epidemiology section, peripheral neuropathy is the most common neurological complication of pSS. It can be present between 20 and 50% of patients when subclinical neuropathy is revealed by a systematic electrophysiological study [47] and clinically from 10 to 32% [6, 14]. In 1962, Kaltreider and Talal [22], described for the first time, the prevalence of neurological involvement in pSS. In this series, 8.3% (*n* = 9) of 109 patients presented neuropathies.

PNS disease includes axonal polyneuropathies (distal axonal sensory and sensorimotor), neuronopathies, mononeuropathies, cranial nerves involvement (mainly trigeminal neuropathy), and autonomic system involvement (Table 1). Axonal polyneuropathies are the most common manifestations of PNS involvement found in 50% of PNS cases [14, 15].

In the series by Gøransson et al. [12], 27% of patients presented peripheral neuropathy and nerve conduction studies were indicative of motor neuropathy in 31% of cases.

### 4.1. Axonal Polyneuropathies

The axonal polyneuropathies are the most frequent clinical presentation of PNS involvement in pSS. It includes distal sensorimotor and sensory polyneuropathies. Clinical manifestations usually start with distal and symmetric sensitive involvement. Large-fiber sensory dysfunction is evidenced by electrodiagnostic studies.

#### 4.1.1. Sensory Polyneuropathy

Distal sensory polyneuropathy is the most characteristic peripheral involvement in pSS [48]. Sensory neuropathy is characterized clinically by sensitive signs on the lemniscal way, with prevalence on the lower limbs. Manifestations include distal paresthesias and evidence of large-fiber sensory dysfunction on examination and electrophysiological studies. In the series described by Mellgren et al. [14], 33 pSS patients with neuropathy were evaluated for neurological examinations, electromyography, and nerve conduction studies. Evaluation also included sural nerve biopsy in 11 patients. Thirty-two percent presented exclusive sensory neuropathy. Mori et al. [11] described 18 patients with painful sensory neuropathy and 36 with sensory ataxic neuropathy from one series of 92 pSS with neuropathy, confirming its high prevalence. This manifestation may be related to skin vasculitis but regularly is not associated with other systemic manifestations of pSS.

#### 4.1.2. Sensorimotor and Motor Polyneuropathy

A mixed sensorimotor polyneuropathy, involving large diameter fibers, most commonly axonal, may be present in pSS. The motor neuron involvement (amyotrophic lateral sclerosis syndrome and anterior horn syndrome) is a rare neurological manifestation in pSS [49] and may be associated with CNS involvement [50].

Another manifestation is the acute motor axonal neuropathy (AMAN), a variant seen in nearly 5% of Guillain-Barré syndrome. More than 60% of AMAN patients have antibodies against ganglioside M1 (GM1) [51, 52]. One case described by Awad et al. [53] showed a patient who developed rapidly fulminant AMAN with anti-GM1 antibodies. Anti-SSA antibodies were also elevated, and sialadenitis was evidenced by minor salivary gland biopsy. This patient responded dramatically to intravenous immunoglobulin (IVIg) treatment.

### 4.2. Sensory Ganglioneuronopathy

Sensory ganglioneuronopathy or sensory ataxic neuropathy produced by posterior spinal roots involvement is manifested as sensory ataxia, and it is characterized by severe impairment of kinaesthetic sensation with no obvious motor involvement [54]. This type of neuropathy may be considered as a subgroup of sensory neuropathy. Physiopathology is probably due to lymphocytic infiltrates on posterior roots and spinal ganglions [11, 24, 54]. In these studies, it has been described lymphocytic infiltrates without vasculitis and degeneration of dorsal root ganglion neuronal cell bodies. Some authors also propose a role of autoantibodies in this manifestation. Among nine patients with pure sensory neuropathy in the study by Delalande [15], four presented clinical and electrophysiological features of sensory ganglioneuronopathy with ataxia. This form of neuropathy is chronic and progressive, occasionally responding to treatment with IVIg [55].

### 4.3. Small-Fiber Neuropathy

Special mention requires the more recent described small-fiber neuropathy in pSS. About 40% of pSS patients experience chronic neuropathic pain with normal electrodiagnostic studies [56–60]. In these cases, quantification of epidermal nerve fiber density in skin biopsy has been validated as a diagnostic tool of small fiber neuropathy [61]. In the biopsy, the intraepidermal nerve fiber density is calculated. In the article published by Fauchais et al. [60], 14 pSSs with chronic neuropathic pain and normal neurological examination were evaluated. Small fiber neuropathy was confirmed by skin biopsy in 13/14 cases. Clinical manifestations were mainly distal burning sensation, dysesthesia, prickling, and allodynia, localized in both hands and feet.

In the outpatient cohort described by Lopate et al. [16], 50% of patients with pSS complained of painful distal paresthesias with evidence of small-fiber sensory loss with normal large-fiber function. Most part of these patients has not been diagnosed before, showing that subclinical or mild neuropathy may be present in pSS and can eventually lead to disability.

The physiopathological mechanism is not well studied. Ischemic and vasculitis processes have been implicated in the small-fiber lesions [62]. Proinflammatory cytokines, such as tumor necrosis alpha (TNF-*α*), have been also implicated, and some clinical improvement has seen with IVIg therapy [63] and anti-TNF-*α* [64] in other clinical conditions. 

Some reports showed that patients who initially presented with a small-fiber neuropathy later developed a sensory ataxic neuropathy [11], suggesting that small-fiber neuropathy is on a continuum with large-fiber sensory neuropathy.

### 4.4. Multiple Mononeuropathy

Similar to multiple mononeuropathy in the context of other AID, this complication is rarely seen in pSS [9, 14]. In the series by Mori el al. [11], 11 of 92 patients with pSS-associated neuropathy (12%) were classified with multiple mononeuropathy. Their clinical evolution is generally faster and more invaliding in pSS compared to other diseases. This complication is associated with cutaneous vasculitis and cryoglobulinemia. The multiple mononeuropathy is mainly produced by ischemic mechanisms [65].

### 4.5. Trigeminal and Cranial Nerves Neuropathies

Often multiple and recurrent cranial nerves neuropathy may be present in pSS. The most common is trigeminal neuropathy, followed by facial and oculomotor nerves involvement [66, 67]. This trigeminal neuropathy presents sensory rather motor involvement. It involves generally the inferior branch of the trigeminal nerve and remains usually clinically unilateral.

Tajima et al. [68] reported the prevalence of trigeminal involvement as high as 50% of patients with cranial nerves compromise. Mori et al. found that 15 of 92 patients (16%) had trigeminal neuropathy with sensory impairment [11]. None presented motor trigeminal involvement. In Delalande serie [15], coclear-vestibular nerve involvement seems to be more frequent (35% of cranial nerve involvement) than trigeminal neuropathy (29%).

### 4.6. Autonomic Neuropathy

In some patients, autonomic neuropathy may be manifested with Adie's pupils, anhidrosis, fixed tachycardia, and orthostatic hypotension [9, 11, 16, 69]. Autonomic symptoms may be explained by both ganglioneuronopathy and vasculitis. Mellgren et al. [14] reported autonomic neuropathy in 6 of 33 patients with pSS (18%). In the series by Andonopoulos et al. [70], autonomic involvement was routinely searched in 32 patients with pSS. Fifty percent of patients presented autonomic symptoms induced by clinical tests. Most of cases have been reported to be mild [71]. Mori et al. reported 3 of 92 patients with sever autonomic neuropathy [11]. Adie's pupil, associated with autonomic involvement in pSS [72], is presumably caused by neuronitis in the ciliary ganglion cells. Antibodies against acetylcholine receptor have been described in patients with pSS and autonomic symptoms [73].

However, other studies have not shown the increased involvement of autonomic system compared to controls. Niemelä et al. [74] performed a complete evaluation of autonomic functions on 30 pSS patients and 30 controls. They showed no differences between the two groups in any of the test, concluding that the prevalence of autonomic dysfunction in pSS is similar to general population.

### 4.7. Polyradiculoneuropathy

Acute of chronic polyradiculoneuropathies have been described in patients with pSS [10, 11]. However, the prevalence in pSS seems to be similar in the clinical, physiopathological, and anatomic context to idiopathic polyradiculoneuropathies.

## 5. Diagnostic of Neurological Involvement in pSS

### 5.1. Cerebrospinal Fluid

CSF may be useful to classify some manifestations. Lymphocytes may be found in some manifestations usually less of 50 cells/mm^3^. In aseptic meningoencephalitis, CSF is abnormal with a higher number of lymphocytes, increased level of proteins, and intrathecal synthesis of gamma globulins [75]. The IgG index is increased during periods of disease activity in up to 50% of cases. CSF is also necessary to the differential diagnosis (i.e., infection, multiple sclerosis). Oligoclonal bands (specifically more than three bands) are highly specific of multiple sclerosis diagnosis. These bands have been reported in about 20 to 25% of pSS compared to more than 90% in MS patients [76–78]. The oligoclonal bands are not stable during the course of the pSS and can disappear after treatment with steroids.

### 5.2. Magnetic Resonance Imaging

MRI abnormalities are common in pSS and usually consist in hyperintense areas in the subcortical and periventricular white matter on T2-weighted and fluid-attenuated inversion recovery (FLAIR) sequences [27, 28]. These lesions are usually less pronounced in pSS than in patients with multiple sclerosis and rarely touch the basal ganglia or the cerebral cortex.

### 5.3. Nerve Conduction Velocity Studies

Motor and sensory nerve conduction velocity studies are tested in the median, tibial, and sural nerve. These values give characteristic patterns about the specific neuropathy, and they can differentiate the two major types: axonal degeneration and demyelinating. Axonal polyneuropathy is the most frequent pattern seen in pSS PNS involvement.

### 5.4. Electromyography

Electromyography patterns such as action potential amplitude twice to normal and an increase in duration of action potential may help to differentiate the neuropathies from myopathies. In pSS, electromyography shows a typical pattern of axonal polyneuropathy, with diminution of sensory amplitudes without latency or conduction velocity involvement. Asymptomatic neuropathies can be found by systematic electromyography test [7].

### 5.5. Sural Nerve Biopsy

Most of the nerve studies in pSS patients with neuropathy have been performed on sural nerve. Mellgren et al. [14] reported vascular or perivascular inflammation of small epineurial vessels in 11 patients with pSS-related neuropathy. In two patients, a necrotizing vasculitis was diagnosed. In this study, axonal degeneration was observed in both sensorimotor and sensory neuropathies. In the study by Griffin et al. [54], most of 12 biopsies showed varying degrees of myelinated fiber loss. Six biopsies had inflammatory infiltrates around epineurial vessels, but necrotizing vasculitis was not evidenced. Cases of multiple mononeuropathy have shown vasculitis in small arteries and arterioles.

### 5.6. Skin Biopsy

Utility of skin biopsy in the diagnosis of pSS-related neuropathy has been described in the section of small-fiber neuropathy.

### 5.7. Neuromuscular Biopsy

The utility of neuromuscular biopsy in pSS-related neuropathy has been evaluated [79]. In the study by Terrier et al., 40 pSS patients with neuropathy underwent neuromuscular biopsy. Pathological results (necrotizing vasculitis in 14 patients and lymphocytic vasculitis in 8) were associated with acute-onset neuropathy, multiple mononeuropathy, and sensorimotor involvement, compared to 18 patients without vasculitis on the neuromuscular biopsy. Necrotizing vasculitis was significantly associated with a better outcome and response to immunosuppressive treatment.

### 5.8. Autonomic Neuropathy Tests

To classify patients with autonomic neuropathy, different test such as Till-table test, gastrointestinal test, thermoregulatory sweat test, or quantitative sudomotor axon reflex test may be used.

## 6. Biological Markers in Patients with pSS and Neurological Manifestations

Anti-Ro and anti-La seem to be less frequent in pSS patients with neurological involvement (40%) compared to patients without neurological manifestations (60% of positivity). Thus, new markers are necessary in pSS to better classify subpopulations of patients with neurological involvement. Some antibodies have been described as potential serological markers of neurological involvement in pSS. However, their useful application is doubtful. IgA and/or IgG anti-alpha-fodrin antibodies in pSS appear to be common in neurological pSS (64.5% of 31 pSS patients with neurological manifestations) [80]. However, this percentage was not different from pSS patients without neurological manifestations. Giordano et al. [81] evaluated IgM and IgG anti-GM1 in 30 pSS patients and its relation with peripheral neuropathy. Anti-GM1 antibodies were present in 12 patients (6 with neuropathy and 6 without), thus showing little help to classify pSS patients with peripheral neuropathy. Antineuronal antibodies have also been described in pSS [82], although their pathological role is unknown.

Anti-GW182 antibodies directed against GW182 protein (a protein located in cytoplasmic structures called GW bodies) have been characterized in autoimmune diseases (mainly in pSS) [83]. In this group, 18 sera of 200 patients (9%) with autoimmune diseases were positive for anti-GW182 antibodies. Interestingly, positive patients had mixed motor and/or sensory neuropathy (*n* = 9), pSS with neurological symptoms (*n* = 3), and 6 patients presented SLE or pSS without neurological manifestations. In conclusion, anti-GW182 antibodies may help to classify patients with autoimmune neurological involvement in different AID.

Of special interest, the antitype 3 muscarinic receptor antibodies have been described in pSS. The IgA isotype may be involved in the pathogenesis of autonomic dysfunction and also may be useful as a novel marker in the pSS diagnosis [84]. Their utility to discriminate patients with neurological involvement has to be tested.  Table 2 summarizes the antibodies in neurological manifestations in pSS patients.

Some other biological markers have been described in neurological involvement in pSS. Among these markers, patients with sensorimotor neuropathy have higher rates of mixed cryoglobulin compared to pSS without neurological manifestations (57% versus 11%), monoclonal gammapathy (71% versus 17%), and NHL (57% versus 3%). On the other hand, patients with sensory neuropathy show lower prevalence of chronic B-cell activation markers (lower prevalence of antinuclear antibodies, anti-SSA, and anti-SSB) [85]. Therefore, these results demonstrate that the pathophysiological mechanism is different according to polyneuropathy type, and the B-cell activation markers can be useful to classify a number of patients with a more severe disease and risk of lymphoproliferation, accompanying some neurological manifestations.

## 7. Treatment of Neurological Manifestations in Sjögren's Syndrome

There is no consensus about the specific treatment of neurological involvement in pSS. Generally, corticosteroid therapy is initiated in patients with either CNS or PNS [15, 86]. CNS involvement is usually treated with high corticosteroid dose. In some cases, response to treatment is exceptional. For example, Caselli et al. [87] showed one patient with dementia who markedly improved after corticosteroid treatment. Concerning the treatment of acute and chronic myelopathies, de Seze et al. [88] showed the tolerance and clinical response of a combination regimen of steroids and monthly cyclophosphamide. Fourteen patients (6 with acute and 8 with chronic myelopathies) were evaluated. Tolerance was good, and nine patients improved clinically (including the total 6 patients with acute myelopathy), three patients remained stable, and the other two patients presented moderate progression. Although randomized studies are necessary, this treatment needs to be considered in patients with progressive disease.

Classically, peripheral neuropathy in patients with pSS responds poorly to treatment [11, 15, 86]. Some groups recommend only treating the symptoms according to the severity. In other patients, immunosuppressive therapy based on corticosteroids, cyclophosphamide, azathioprine, and even plasmapheresis has shown only mild success [89–91].

In the series reported by Terrier et al. [79], patients with necrotizing vasculitis have a better response to immunosuppressive treatment, mainly with cyclophosphamide (71% of patients with necrotizing vasculitis showed good response compared to 25% of patients with lymphocytic vasculitis). Griffin et al. reported a treatment based on corticosteroids and associated in some cases with azathioprine, intravenous cyclophosphamide or plasma exchanges [54]. Only one patient with a relapsing course responded to corticosteroid treatment. Mori et al. suggested that corticosteroids are suitable for multiple neuropathy and multiple cranial neuropathy [11].

IVIg has been also reported as a good therapeutic option in some painful sensory neuropathy cases [92] and in radiculoneuropathy. In a recently series of 19 pSS patients with peripheral neuropathy, intravenous immunoglobulin treatment was evaluated [93]. In this study, 8 patients (42%) showed a decrease of the disability Modified Rankin Scale, corresponding to a clinical improvement. Patients with sensorimotor or nonataxic sensory neuropathy were markedly improved compared to patients with ataxic neuropathy (2/9). The authors concluded that clinical benefits of IVIg treatment depend on the specific clinical subtype.

Caroyer et al. [94] showed improvement in sensory ganglioneuronopathy treated with infliximab. However, no controlled trials have shown efficacy of infliximab or others anti-TNF*α* in pSS-related neuropathy.

Rituximab, an anti-CD20 antibody, may be useful in systemic complications in pSS patients [95, 96] and in some cases of refractory neuropathy. Recently, Mekinian et al. [97] reported 17 patients with pSS and PNS involvement treated with rituximab. Neurological improvement was observed in 11/17 patients (65%) at three months. Best results were observed in patients with cryoglobulinemia or vasculitis-related PNS involvement (9/10 patients improved).

The benefits from treatment with oromucosal IFN-*α* in pSS have been reported by several groups [98–101]. Due to possible effects on sicca symptoms, Yamada et al. [102] reported three cases of pSS-associated neuropathy treated with oral IFN-*α* (two patients with sensory ataxic neuronopathy and one patient with axonal sensorimotor neuropathy with demyelinating features). All three patients responded well to IFN-*α*, improving the neurological symptoms. Sicca symptoms, antibodies titres, and focus score of salivary gland biopsy were also improved. However, the mechanisms whereby IFN-*α* induces neurological improvement in pSS are uncertain.

In conclusion, neurological manifestations are common in pSS and often precede the diagnosis. The accurate prevalence of these manifestations is difficult to assess, because the heterogeneity of the series. The pathogenic mechanisms responsible for most forms of neurological involvement in pSS remain unknown, but vascular, ischemic, and immunological mechanisms have been described. Controlled and population-based trials are necessary to better characterize the neurological manifestations in pSS and their therapeutic response.

## Figures and Tables

**Table 1:** Neurological manifestations in primary Sjögren's syndrome.

Peripheral disorders	Central disorders
Axonal polyneuropathies (i) Symmetric pure sensory peripheral neuropathy (ii) Symmetric sensorimotor peripheral neuropathy	Focal (i) Seizures (ii) Movement disorders (iii) Cerebellar syndrome (iv) Optic neuropathies (v) Pseudotumor lesions (vi) Motor and sensory loss

Sensory ganglioneuronopathy	Multifocal disease (i) Cognitive impairment (ii) Encephalopathy (iii) Dementia (iv) Psychiatric abnormalities (v) Aseptic meningoencephalitis

Motor neuropathy	Spinal cord dysfunction (i) Chronic progressive myelopathy (ii) Lower motor neuron disease (iii) Neurogenic bladder (iv) Acute transverse myelitis

Small-fiber neuropathy	Progressive-multiple sclerosis-like syndrome

Multiple mononeuritis	Central nervous system vasculitic involvement

Trigeminal and other cranial nerves neuropathies	

Autonomic neuropathies	

Demyelinating polyradiculoneuropathy	

**Table 2:** Antibodies in neurological manifestations of primary Sjögren's syndrome.

Antibody	Clinical association	Reference
Anti-SSA and anti-SSB	Most of studies show lower prevalence of anti-SSA and anti-SSB antibodies in pSS with neurological involvement. In one series, patients with nonataxic sensory neuropathy had lower prevalence of anti-SSA (40% versus 72%) and anti-SSB (15% versus 41%).	Sene et al. [85]

Anti-SSA	This paper showed that anti-Ro antibodies were positive in 48% of patients with CNS compared to only 24% of all patients with pSS. However, the anti-SSA antibodies were detected by double immunodiffusion and not by ELISA.	Alexander et al. [18]

Anti-alpha fodrin (IgA and IgG)	These antibodies are common patients in pSS. However, there are not differences between patients with or without clinical neurological involvement.	De Seze et al. [88]

Anti-GM1 (IgM and IgG)	No differences between pSS patients with or without neurological involvement.	Giordano et al. [81]

Antineuronal antibodies	In a large series of patients with neurological disorders (*n* = 882), these antibodies were detected in patients with pSS and neurological involvement, although the specificity has to be defined. Antiganglion neuron antibodies have been also reported.	Murata et al. [25], Vianello et al. [82]

Anti-GW182	Detected in patients with mixed motor and/or sensory neuropathy without pSS and also in neurological involvement in pSS patients.	Eystathioy et al. [83]

GM1: ganglioside; GW182: protein located in cytoplasmic structures called GW bodies; CNS: central nervous system; pSS: primary Sjögren syndrome.
